# Germination Biology of Two Invasive *Physalis* Species and Implications for Their Management in Arid and Semi-arid Regions

**DOI:** 10.1038/s41598-017-17169-5

**Published:** 2017-12-05

**Authors:** Cumali Ozaslan, Shahid Farooq, Huseyin Onen, Selcuk Ozcan, Bekir Bukun, Hikmet Gunal

**Affiliations:** 1 Department of Plant Protection, Dicle University, Diyarbakir, Turkey; 2 Department of Plant Protection, Gaziosmanpasa University, Tokat, Turkey; 3 Pistachio Research Station, General Directorate of Agricultural Research and Policies, Gaziantep, Turkey; 4 Department of Soil Science and Plant Nutrition, Gaziosmanpasa University, Tokat, Turkey

## Abstract

Two Solanaceae invasive plant species (*Physalis angulata* L. and *P. philadelphica* Lam. var. *immaculata* Waterfall) infest several arable crops and natural habitats in Southeastern Anatolia region, Turkey. However, almost no information is available regarding germination biology of both species. We performed several experiments to infer the effects of environmental factors on seed germination and seedling emergence of different populations of both species collected from various locations with different elevations and habitat characteristics. Seed dormancy level of all populations was decreased with increasing age of the seeds. Seed dormancy of freshly harvested and aged seeds of all populations was effectively released by running tap water. Germination was slightly affected by photoperiods, which suggests that seeds are slightly photoblastic. All seeds germinated under wide range of temperature (15–40 °C), pH (4–10), osmotic potential (0 to −1.2 MPa) and salinity (0–400 mM sodium chloride) levels. The germination ability of both plant species under wide range of environmental conditions suggests further invasion potential towards non-infested areas in the country. Increasing seed burial depth significantly reduced the seedling emergence, and seeds buried below 4 cm of soil surface were unable to emerge. In arable lands, soil inversion to maximum depth of emergence (i.e., 6 cm) followed by conservational tillage could be utilized as a viable management option.

## Introduction

Two invasive plant species of Solanaceae family, i.e., *Physalis angulata* L. (cutleaf groundcherry) and *P. philadelphica* Lam. var. *immaculata* Waterfall (Mexican groundcherry) are distributed in several parts of the world. Although both plant species are used as food crops and have many medicinal benefits, these have also been reported as noxious/invasive weeds of cotton, corn and soybean in several parts of the world^1–3^. *P. angulata* was firstly reported in Turkey during 2000^4^, whereas first record of *P. philadelphica* dates back to 2002^5^. Both species are listed as “invasive” in Turkey^6,7^, co-occur in the distribution range and infest different field crops including cotton, maize, cucumber, tomato, pepper and olive orchards in Southeastern Anatolia (SEA) region of the country^8^. The SEA region have an arid to semi-arid climate with hot summers^9,10^. The evaporative demands of crops are met by irrigation in the region. Inadequate infrastructure of drainage^11^ and excessive use of irrigation water coupled with high evaporation have raised soil salinity in the region^11^. Farooq *et al*.^8^ indicated irrigation as primary, and soil pH, and electrical conductivity as secondary invasion drivers of both species in the region.

*P. angulata* and *P. philadelphica* produce enormous amounts of seeds even under adverse environmental conditions^10^, which are readily deposited to soil seed bank^12,13^. Management of these plant species, due to their annual nature and enormous seed production capability is difficult without the use of herbicides^12,14^. However, recorded lower control of some herbicides^1^ indicates the evolution of herbicide resistance. Thus, adoption of alternative sustainable management practices is necessary. Germination biology is important to develop management strategies against weeds and invasive plant species^15^. Germination under wide range of environmental conditions increases the chances of successful invasion^16–18^. The knowledge of germination biology could facilitate management of invasive plant species either by suppressing or stimulating the germination when seedlings can be managed successfully^19^. The differences in germination and emergence patterns of different populations are also of great significance for inferring the further range expansion at regional scales^18^.

Variations in seed traits among and within populations are among the key factors responsible for establishment and persistence of invasive plant species^20–22^. Seed traits are governed by many factors including genetic variation and environmental conditions prevailing during seed development^22,23^. These factors collectively determine the persistence (dormancy), germination and dispersal or mortality^23–26^ of seeds produced by a plant species. The seeds of different individuals within a population of the same species show variations in seed germination biology, including dormancy and receptiveness of dormancy-breaking factors^27^. Moreover, seeds collected from different sites, years, altitude gradients and habitats also exhibit differences in seed germination biology due to inherent environmental conditions^23,28–30^.

Seed germination in natural and cultivated habitats is significantly influenced by several environmental factors such as temperature, light, moisture, pH, salinity and seed burial depth^19,24,31,32^. Temperature influences the germination by regulating the enzyme activities and promote/inhibit the hormone synthesis that promote dormancy/germination^33^. Light requirement of weeds/invasive plant species determine their emergence patterns with reference to seed burial depth^24,33^. For example, seeds requiring light for germination will not emerge when buried deep in the soil^24^. Besides, seeds present in the soil seed bank at various depths exhibit different germination response with respect to variation in moisture, temperature, light, EC and pH.

Both *Physalis* species are widely distributed in arid and semi-arid regions of Turkey and started to incur losses in crop production^1,8^, however no information is available on their germination biology. Both plant species are naturally found in tropical parts of the world^34^ and a little information is present for germination requirements of *P. angulata* ^13^. However, the species in Turkey are observed in various habitats of different climatic zones (especially arid and semi-arid regions) with different elevation gradients^6,7^. Germination biology of these species could give valuable insights on their possible adaptations strategies for persisting in arid and semi-arid regions, could facilitate to assess their further spread potential and help in devising successful management strategies.

The current study was conducted to infer the dormancy patterns, effects of temperature, light, water stress, pH and salt stress on germination, and effect of seed burial depth on seedling emergence of different populations of *P. angulata* and *P. philadelphica*. The germination behavior will give theoretical and practical information about their possible adaptation strategies in arid and semi-arid region, range expansion potential and management. It was hypothesized that; i) populations arising from higher altitude will show variations in dormancy and optimum light and temperature requirements for germination compared to lower altitudes, ii) both co-occurring plant species will probably show similar germination behavior under similar environmental conditions, iii) increasing pH, water stress and salt stress will decrease germination and iv) increasing seed burial depth will decrease seedling emergence.

## Results

**Figure 1:**
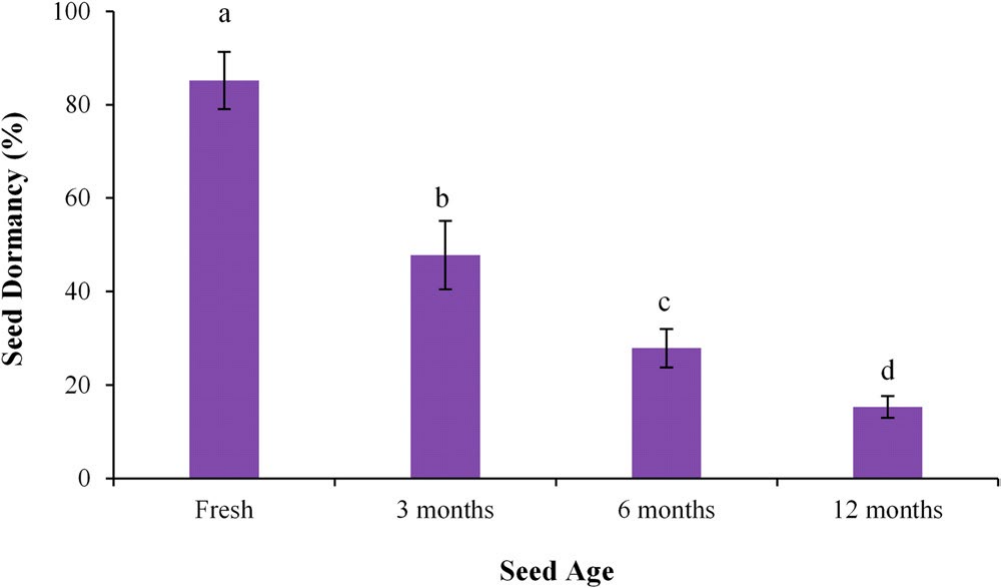
Effect of seed age on seed dormancy of different populations of *Physalis angulata* and *P. philadelphica* arising from various elevation gradients and habitats. Any two means sharing different letters are significantly different from each other. The vertical bars represent standard errors of means.

**Figure 2:**
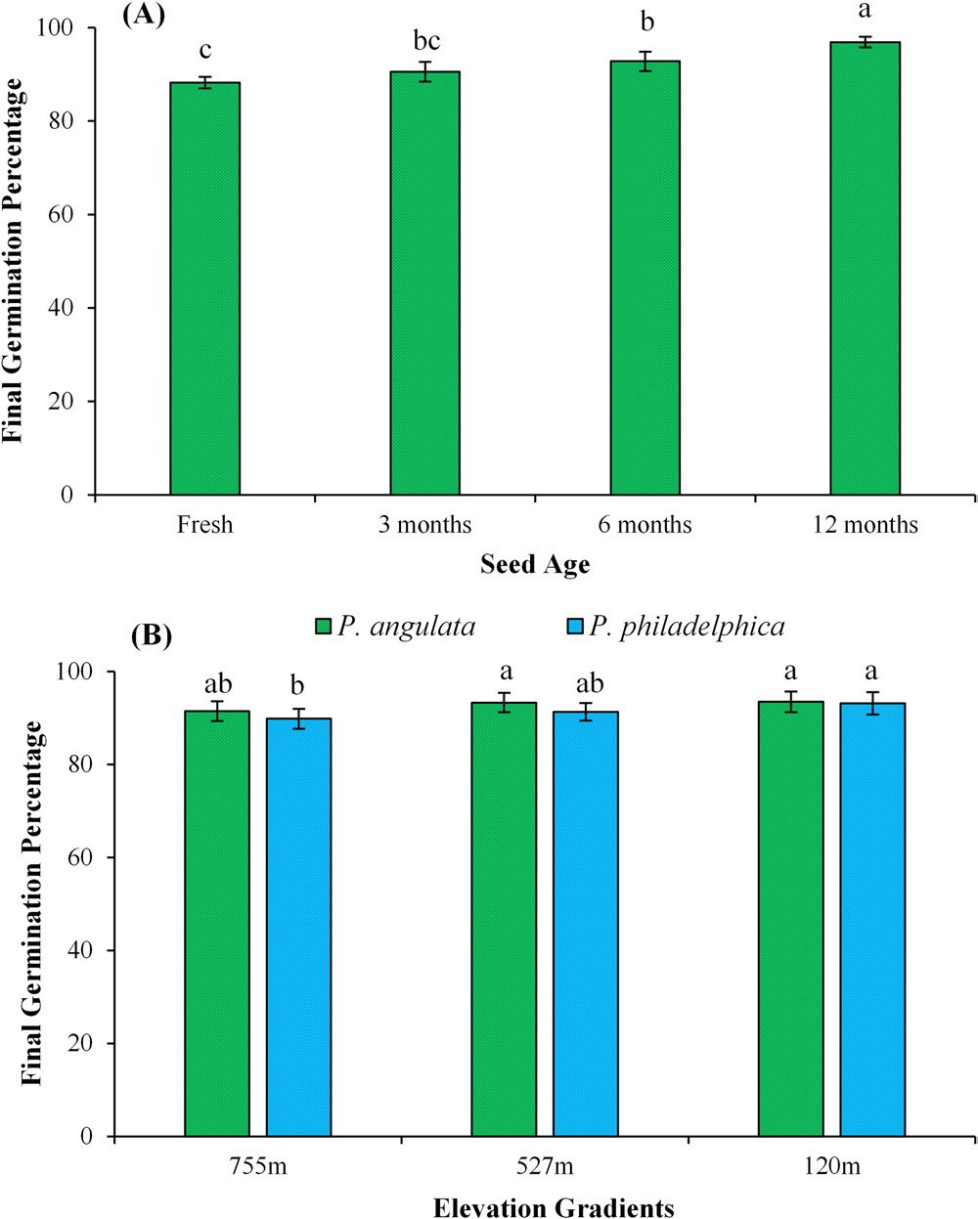
Effect of seed age (**A**) and invasive plant species × populations’ interactions (**B**) on seed dormancy release (seeds kept under running tap water for 24 hours) of different populations of *Physalis angulata* and *P. philadelphica* arising from various elevation gradients and habitats. Any two means sharing different letters are significantly different from each other. The vertical bars represent standard errors of means.

Seed age significantly affected the seed dormancy level of different populations of both plant species (Table S1). Freshly harvested seeds of all populations of both plant species were highly dormant. Seed dormancy was released with increasing age of seeds as freshly harvested seeds had 85.22% seed dormancy, while it was reduced to 15.33% in 12 months old seeds (Fig. 1). The seed dormancy of fresh and aged seeds of both plant species was effectively released by running tap water. Seed germination was progressively increased by running tap water with increasing age of the seeds (Fig. 2a). The interactive effect of plant species and their populations on final germination percentage of seeds kept under running tap water was also significant (Table S2). Seeds collected from higher elevation gradient had slightly higher seed dormancy level compared to the seeds collected from lower elevation gradients (Fig. 2b).

**Figure 3:**
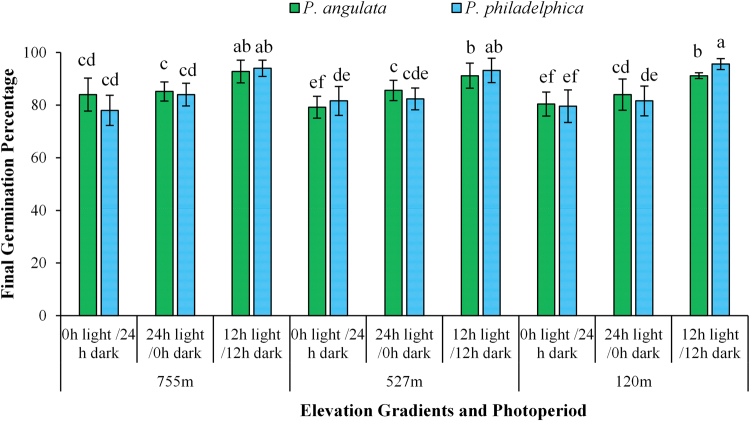
Effect of different photoperiods on germination of *Physalis angulata* and *P. philadelphica* populations collected from various elevation gradients and habitats. Any two means sharing different letters are significantly different from each other. The vertical bars represent standard errors of means.

Seed germination was significantly influenced by photoperiods, plant species × photoperiods and plant species × population’s × photoperiods’ interactions (Table S3). The seeds of both plant species incubated in 12 h light and 12 h dark period exhibited the highest germination, while continuous dark and continuous light treatments had lower final germination percentage for all populations of tested plant species (Fig. 3). Similarly, the seeds of populations collected from higher elevation gradient had lesser final germination percentage compared to those collected from lower elevations (Fig. 3).

**Figure 4:**
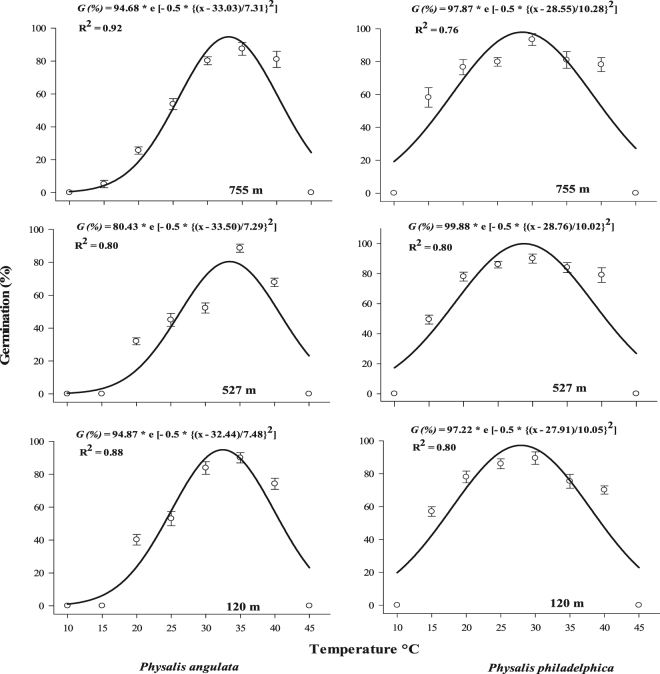
Effect of different constant temperature regimes on final germination percentage of different populations of two invasive *Physalis* species collected from varying elevation gradients and habitats. The lines represent three parametric Gaussian model fitted to the final germination data at 30 days after start of the experiment. The vertical bars represent standard errors of means.

Germination was significantly (*p* ≤ 0.01) affected by individual and interactive effects of plant species, populations and experimental treatments (i.e., different temperatures, pH, water stress and salt stress levels (Tables S4–S7). Similarly, seedling emergence was also significantly influenced by individual and interactive effects of plant species, populations and seed burial depths (Table S8). *P. philadelphica* seeds were able to germinate over wide range of temperature regimes (>50% germination under 15 to 40 °C) compared with *P. angulata* (>50% germination under 25–40 °C) (Fig. 4). *P. angulata* had higher temperature requirement (optimum temperature for germination) for maximum germination (33.03, 33.50 and 32.44 °C for populations collected from 755, 527 and 120 m altitude, respectively). Contrastingly, *P. philadelphica* required lower temperature for maximum germination (28.55, 28.76 and 27.91 °C for populations collected from 755, 527 and 120 m altitudes, respectively) (Fig. 4).

**Figure 5:**
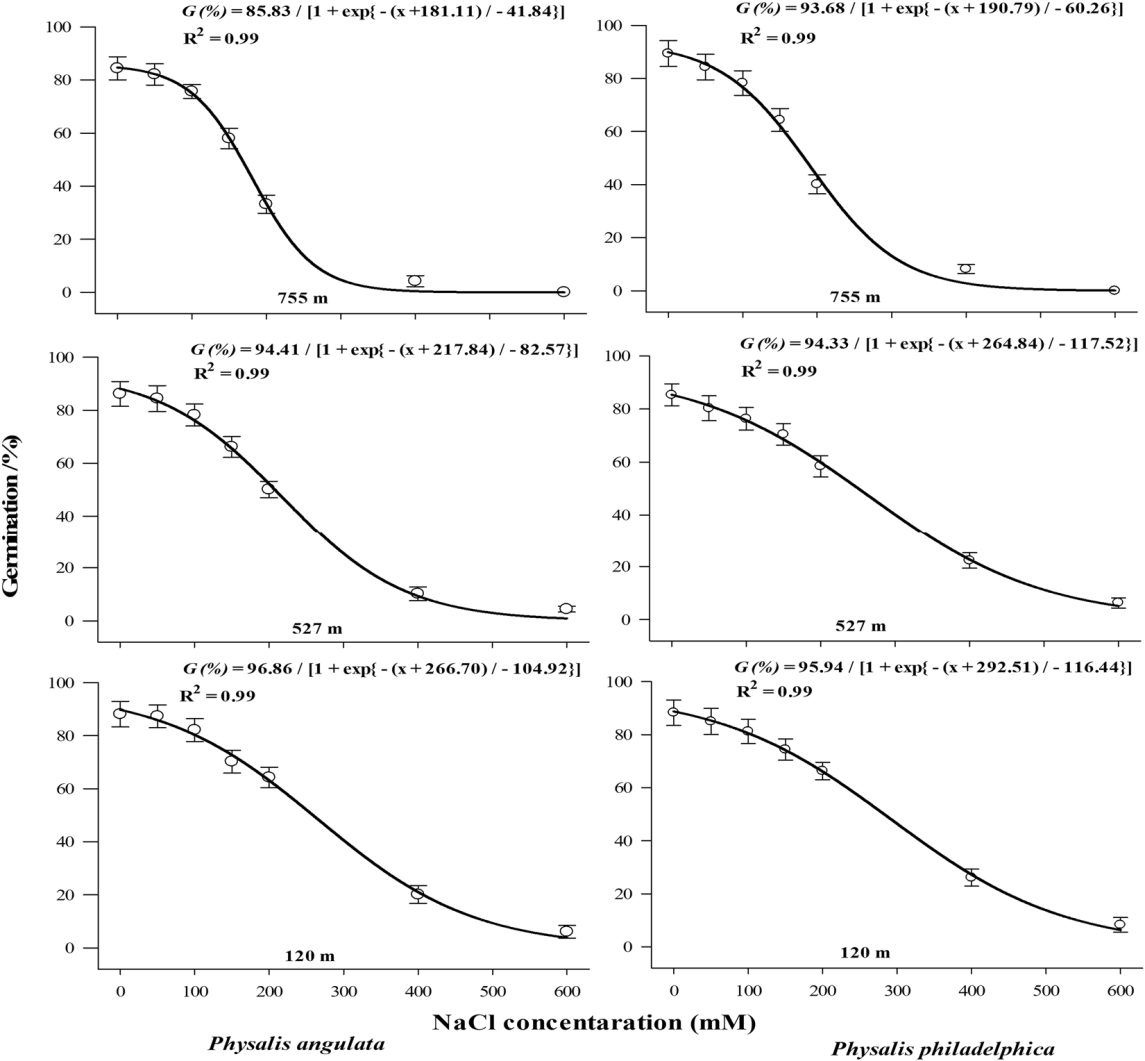
Effect of varying pH levels on final germination percentage of different populations of two invasive *Physalis* species collected from different elevation gradients and habitats. The lines represent three parametric Gaussian model fitted to the final germination data at 30 days after start of experiment. The vertical bars represent standard errors of means.

Seeds of tested species were capable of germinating under a broad range of pH (5 to 10). However, highly acidic or alkaline pH considerably reduced seed germination (Fig. 5). Populations collected from higher altitudes required pH near to neutral for peak germination (6.98 and 7.50 for *P. angulata* and 7.16 and 7.29 for *P. philadelphica* populations collected from 755 and 527 m, respectively). Interestingly peak germination in lower altitude (120 m) populations was observed under slightly alkaline pH (7.93 and 7.98 for *P. angulata* and *P. philadelphica*, respectively) (Fig. 5).

**Figure 6:**
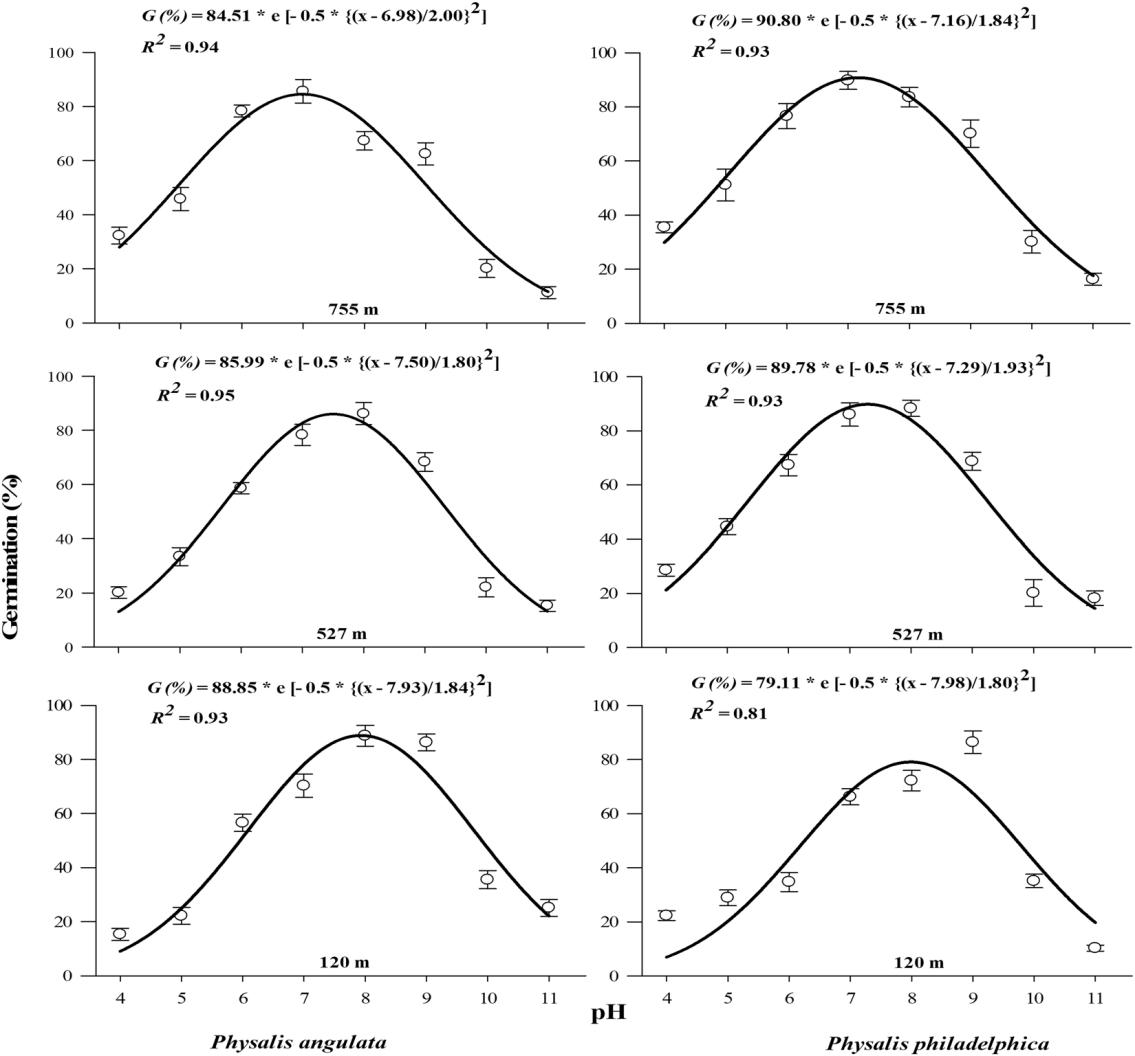
Effect of different osmotic potentials (water stress) on final germination of different populations of two invasive *Physalis* species collected from different elevation gradients and habitats. The lines represent three parametric sigmoidal function fitted to model the final germination data at 30 days after start of the experiment. The vertical bars represent standard errors of means.

**Figure 7:**
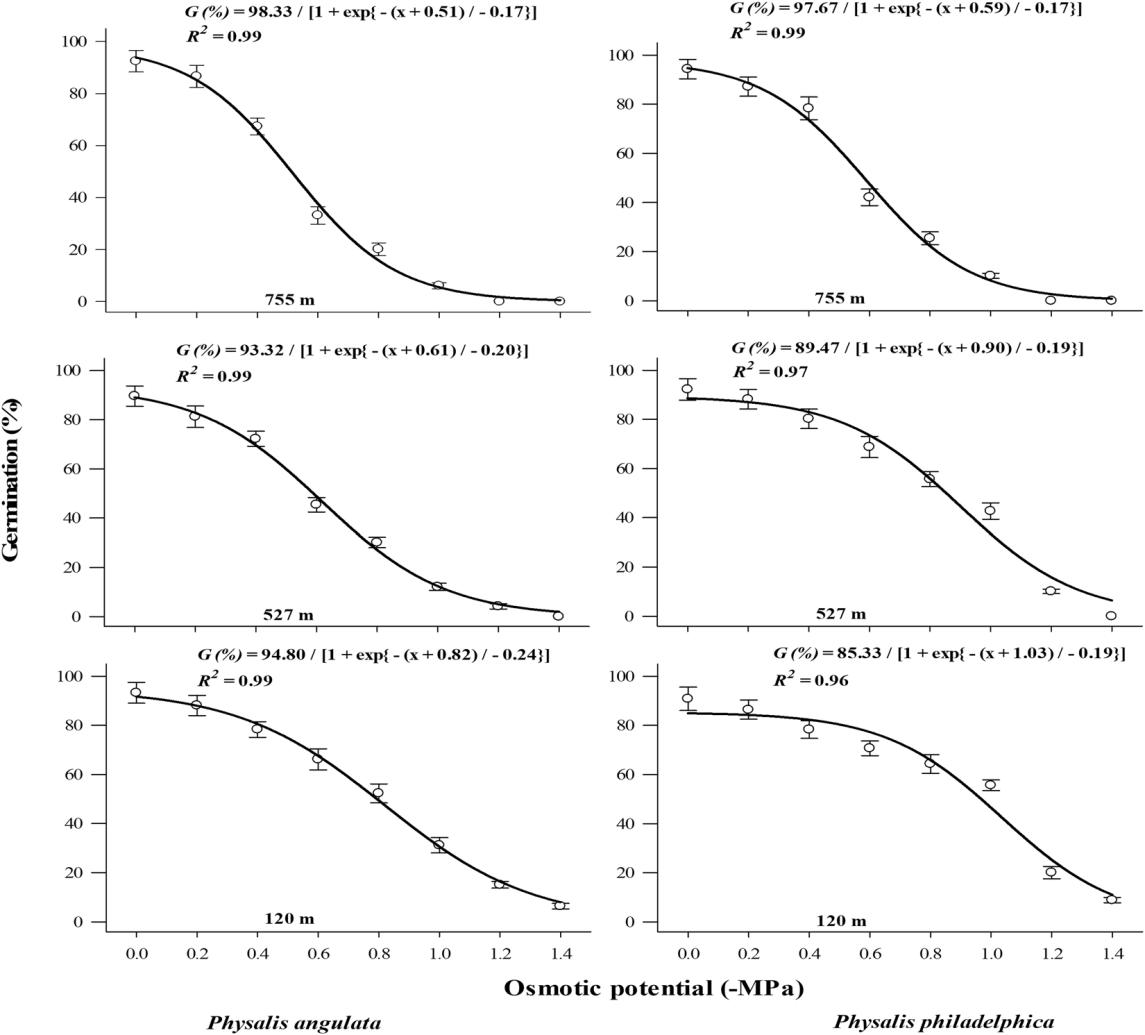
Effect of different NaCl concentrations (salt stress) on final germination percentage of different populations of two invasive *Physalis* species collected from different elevation gradients and habitats. The lines represent three parametric sigmoidal function fitted to model the final germination data at 30 days after start of experiment. The vertical bars represent standard errors of means.

*P. philadelphica* seeds germinated under broad range of osmotic potentials (>50% germination under −0.59 to −1.03 MPa). However, germination of *P. angulata* seeds was reduced under higher (>50% germination under −0.51 to −0.82 MPa) osmotic potentials (Fig. 6). Populations arising from higher altitudes were lesser tolerant to water stress (osmotic potential required to inhibit 50% of maximum germination was low) compared to those collected from low altitude (Fig. 6). Similar to water stress, *P. philadelphica* seeds proved more tolerant to salinity (higher salinity levels were required to retard 50% of the maximum germination) compared with *P. angulata* (Fig. 7). Similarly, lower altitude populations were more tolerant to higher salinity levels compared with populations collected from higher altitudes (Fig. 7).

**Figure 8:**
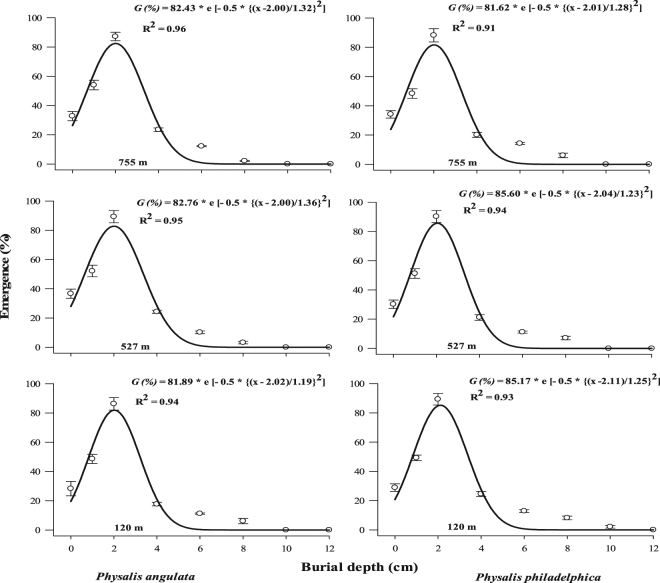
Effect of seed burial depth on seedling emergence percentage of different populations of two invasive *Physalis* species collected from different elevation gradients and habitats. The lines represent three parametric Gaussian model fitted to the final germination data at 40 days after start of experiment. The vertical bars represent standard errors of means.

Seedling emergence was initially increased up to 2 cm (maximum seedling emergence was recorded at this depth) and sudden decline in seedling emergence was recorded for both plant species (Fig. 8). More than 80% of seedlings of tested species were unable to emerge when seeds were buried below 6 cm soil depth (Fig. 8).

## Discussion

Dormancy and germination are considered as important adaptive traits for successful establishment of invasive plant species. Higher dormancy helps to persist in soil seed bank for longer periods, whereas germination under wide range of environmental conditions enables the plant species to adapt diverse ecological conditions. Therefore, higher germination and seedling recruitment have been recognized among the major factors promoting naturalization success of invasive plant species^18,23,35^.

In the current study, freshly harvested seeds of different populations of *P. angulata* and *P. philadelphica* exhibited equal level of dormancy (unlike as hypothesized), although collected from different altitudinal gradients and habitats. Seed traits, such as dormancy are highly dependent on the environmental conditions faced by the maternal plants prior to seed filling and ripening^22^. The species tested in the current study are probably in their naturalization phase as these have been recently observed in the country^4,5^. Thus the tested species invest all resources on seedling recruitment as it promotes establishment and subsequent spread of invasive plant species^18,35^. Since both species have recently been reported in the country, long term climatic conditions have probably not influenced the dormancy traits^6,7^. Although several studies indicated that different populations of same species might show different levels of seed dormancy^23,27^, some contrasting reports also revealed that different populations of some species have equal levels of dormancy and seed germination^36^.

Seed dormancy level of all populations of both species was linearly decreased with increasing seed age. Running tap water effectively released dormancy of both freshly harvested and aged seeds of all populations of both species. The seeds probably exhibit dormancy due to toxic substances present on the seeds immediately after harvest and running tap water flushed these substances, thus released dormancy. Irrigation is frequently practiced for agricultural production in SEA region, therefore irrigation water is thought to be sufficient for releasing seed dormancy under natural conditions in the currently invaded areas.

Light is inherent requirement for germination of some weed species, while others do not require light at all for germination^31,32,37,38^. Seeds of different populations of both species proved slightly photoblastic. It has also been reported earlier that *P. angulata* have no strict light requirements for seed germination^13^. The slightly photoblastic nature of the seeds indicates that seedlings could emerge from different seed burial depths which explains the distribution of both species in diverse habitats in the country^6–8^.

Temperature influences germination by regulating enzyme activities and promotes/inhibits the hormone synthesis required to promote dormancy/germination^33,39^. Different populations of the tested *Physalis* species responded differently to various constant temperatures (Fig. 4). *P. angulata* germinated under 25 to 40 °C with highest germination under 35 °C. Whereas, *P. philadelphica* not only germinated under wide range of temperatures (15 to 40 °C), but also exhibited maximum germination under 25–30 °C. The results are controversial to findings of Bell and Oliver^13^, who reported that *P. angulata* seeds failed to germinate under constant temperatures. The differences in temperature requirements are owed to their differential adaptation potential. The adaptations for germination under diverse environmental conditions are of great ecological significance indicating the potential of invasive plant species to invade more areas. Several studies have been conducted to determine the optimum temperature requirements of different weeds and invasive plant species^24–26,31,32,37,38^. The findings on the germination adaptations of both species to persist under arid and semi-arid regions are novel.

The distinct temperature requirements of different populations are also quite interesting. Both species share the same origin and currently distributed in the same region in Turkey^6,7^, however, undergone differential adaptations for temperature requirements. The results indicate that *P. philadelphica* is able to withstand harsher environmental conditions compared with *P. angulata*. The results also suggest that *P. philadelphica* have higher range expansion potential to even warmest regions of the country, while *P. angulata* could be limited to only slightly warmer areas.

Seeds of both plant species were able to germinate under wide range of water and salinity stresses. The populations collected form lower altitude and agricultural habitats proved more resistant to higher levels of water and salinity stress compared to those from higher altitudes. The germination potential of different populations indicates that they have distinct advantage of range expansion to even marginal habitats. A large portion of Turkey have arid and semi-arid climate along with relatively high salinity^9,11^, which is suspected to be under the invasion risk. Higher germination under elevated levels of water and salinity stress is probably related to higher tolerance of low altitude populations to low rainfall (Table 1) and high salinity levels^11^ compared to high altitudinal populations. However, both plants species, especially *P. philadelphica*, not only germinate under higher water stress and salinity levels, but also capable of growing and producing enormous amounts of seeds^2,10,13^. Therefore, the plant species are expected to create severe economic losses and ecological problems in the region as the invasion further expands to the non-infested areas.

**Table 1:** Background information on different populations of *Physalis angulata* L. and *P. philadelphica* Lam. var. *immaculata* Waterfall.

Site	Habitat	Longitude (E)	Latitude (N)	Altitude (m)	AI	AP (mm)	PET (mm)
**1**	Agriculture (Pepper)	33.43	36.54	120	0.34	404.19	1181.07
**2**	Abandoned land	41.47	37.83	527	0.47	556.71	1172.25
**3**	Agriculture (Tobacco)	38.42	37.85	755	0.56	613.13	1100.33

AI = Aridity index, AP = Annual precipitation, PET = Potential evapotranspiration.

Irrigation, salinity and fine texture of soils have been reported as main invasion drivers of both plant species in the region^8^. Due to inadequate drainage infrastructure and excessive use of irrigation water, soils are poorly drained and hold water for long time which naturally releases seed dormancy. Hence suitable irrigation systems (such as drip irrigation) which do not provide moisture over all field could be used to control the germination of both species. Both species could outcompete natural vegetation due to the high tolerance to salinity and drought^10^, thus effective management practices are inevitable to combat with them.

Different populations of both species germinated under wide range of pH indicating that they have adapted to diverse range of soil environments. Maximum germination under alkaline pH indicates that agricultural populations of both species have adapted to existing pH in the region and could invade areas of higher soil pH as suggested in the earlier studies for other weeds and invasive plant species^33,38,40^. Excessive use of fertilizers and irrigation water in the region raise EC and soil pH^11^, therefore alternative irrigation systems and sustainable use of fertilizers warrant stronger justification for suppressing both species. Like in temperature, water and salinity stress experiments, *P. philadelphica* seeds had higher germination rate under wider range of pH compared to *P. angulata*. This also supports the above findings that *P. philadelphica* could be more problematic in future.

Seed burial depth initially increased the seedling emergence up to 2 cm and then a sharp decline was observed for deeper seed burial depths. The poor soil and seed contact and low water imbibition are the possible reasons of low emergence observed in the seeds placed on soil surface^24^. Seeds proved slightly photoblastic and it was expected to emerge even from deeper soil layers. However, emergence was severely retarded beyond 2 cm depth (Fig. 8). Lower emergence of several weed species due to the higher burial depth have been reported^24,38,41,42^. As seeds proved slightly photoblastic and are of smaller size, lack of energy to push the seedling from increased burial depth is probably the reason of low emergence in the current study^42,43^.

Seeds of *P. philadelphica* progressively lose vigor and germination ability at a rate of 9% annually^44^. It seems that soil inversion by tillage to a maximum depth of emergence could be used as an efficient management tool to combat with both species. However, mold board plow (conventional tillage) turns over topsoil (bringing buried weed seeds to the top), which is frequently practiced in the region for preparing seedbed especially for cotton crop. Cotton is the most infested crop in the SEA^8^, therefore seeds buried can again come to soil surface with tillage in subsequent growing seasons. The results of seed burial experiments provided two distinct directions for management of the species. The first option is to bury the seeds deep enough in soil and practice conservational tillage in the subsequent years. Conservational tillage along with effective management of emerging seedlings will deplete the soil seeds banks in the long run. The second option is to practice only shallow tillage and manage the emerging seedlings through integrated weed management approach.

## Conclusion

The results of the current study suggest that both plant species are in naturalization phase and exhibit no differences for seed dormancy trait. However, both species have undergone extensive adaptations for germination to persist under wide range of environmental conditions. The country has a large portion with arid and semi-arid climate along with high salinity, therefore both plant species could expand their range to even these marginal habitats. *P. philadelphica* is expected to invade more areas compared with *P. angulata* in future. Soil inversion to bury the seeds to a maximum depth of emergence followed by conservational tillage practices and managing emerging seeds could be used as a viable management tool to reduce the densities and soil seed bank of both species in long run. Similarly, adoption of an alternative irrigation system could simply suppress the germination of both species as well. The results of the current study necessitate the studies on developing alternative, effective management strategies against both weeds under different tillage and irrigation systems.

## Methods

### Seed collection

The seeds were collected from different populations of *P. philadelphica* and *P. angulata* distributed at various elevation gradients and habitats in South (Mersin) and South eastern Anatolia (Adiyaman and Batman provinces) regions of Turkey. The mature berries of both species were harvested and brought to laboratory, dried, cleaned and used in the experiments. The information related to habitat and prevailing environmental conditions at the seed collection sites are provided in Table 1.

### General procedure for germination

Freshly harvested seeds (except dormancy experiment) were used in all experiments and the dormancy was released by keeping seeds under running tap water for 24 hours. The seeds were then placed on two layers of moistened Whatman no.1 filter paper in 9 cm Petri dishes. The filter paper layers were initially moistened with 4 ml deionized water or respective treatment solution and then moistened according to requirements. In all experiments, 5 replicates of 50 seeds placed in Petri dishes were incubated at 30 °C, 12 h photoperiod and 60% relative humidity. Experiments were conducted in completely randomized design and place of Petri dishes was changed every day. The germinated seeds were counted daily (except photoperiod experiment to exclude the effect of light on seed germination) for 30 days taking radical protrusion (2 mm visible) as criterion. Germinated seeds were removed from the Petri dishes. The germination for photoperiod experiment was observed at the end of experiment. Germination percentage was computed 30 days after initiation of the experiments and used in analysis. Experiments were repeated over time (two experimental runs for each type of experiment) for validation of results.

### Experiment 1: Seed dormancy level and seed dormancy release

Freshly collected seeds of all populations of both species were dormant (see results section for details). Therefore, effect of seed age and running tap water on seed dormancy release was tested. Seeds of different age (fresh, 3, 6 and 12 months old) were incubated in light to infer the levels of seed dormancy. For running tap water treatment, seeds were wrapped in a thin cloth and kept under running tap water for 24 hours. The seeds were then surface dried and germination test was completed as described above.

### Experiment 2: Effect of photoperiod on seed germination

Germination of freshly harvested seeds all populations of both species was observed under three different photoperiods (continuous dark, continuous light and 12 hours alternating light and dark) to determine the effect of light on germination. The light was provided by cool, white fluorescent lamps, at 350 µEm^−2^ s^−1^ intensity, whereas the Petri dishes were wrapped in four layers of aluminum foil for creating complete dark.

### Experiment 3: Effect of temperature on seed germination

To determine the effects of temperature on germination, freshly harvested seeds of different populations were germinated under a wide range of constant temperatures (10, 15, 20, 25, 30, 35, 40 and 45 °C). The temperature regimes were constant (no day and night variation) throughout the experiment duration. The higher temperature regimes were included in the experiment because of high temperature prevailing in South eastern Anatolia region, Turkey^9^ during the growth season of both plant species.

### Experiment 4: Effect of water stress on seed germination

Freshly harvested seeds of all populations of both plant species were incubated with osmotic potentials of −0.2, −0.4, −0.8, −1.0, −1.2 and −1.4 MPa along with a control treatment (0 osmotic potential, only distilled water) to test the effect of water stress on germination. The osmotic potential solutions were prepared by dissolving polyethylene glycol 8000 in distilled water as described earlier^45^.

### Experiment 5: Effect of salt stress on seed germination

To test the effect of salt stress on germination of different populations of both *Physalis* species, freshly harvested seeds were incubated in sodium chloride (NaCl) solution of different concentrations (50, 100, 150, 200, 400 and 600 mM). The experiment also had a control treatment (only distilled water) for comparison.

### Experiment 6: Effect of pH on seed germination

To assess the effects of pH on germination of both species, freshly harvested seeds of different populations were germinated under wide range of pH (4.0, 5.0 and 6.0 for acidic medium), (7.0 as neutral medium) and (8.0, 9.0, 10.0 and 11.0 as alkaline medium). The solutions were prepared as described in previous study^46^.

### Experiment 7: Effect of seed burial depth on seedling emergence

A greenhouse experiment was conducted to test the effect of seed burial depth on seedling emergence of both plant species by placing 50 seeds on the soil surface (0 cm burial depth) or in the soil filled in 15 cm diameter plastic pots at various depths. Seeds were buried at various depths (1, 2, 4, 6, 8, 10 and 12 cm) by covering the seeds with soil to achieve the respective seed burial depth. A seed was considered as “emerged” when cotyledon was easily visible. The pots were initially irrigated with overhead sprinkler, while sub-irrigation was applied at later stages of experiment. The experiment was carried out for 40 days starting from the day of seed burial.

### Statistical analyses

The collected data were analyzed in four steps. In the first step, the difference between experimental runs were tested using Paired T test. No significant differences were observed in experimental runs, therefore data of both experimental runs were combined for further processing. The data of each type of experiment were subjected to analysis of variance (ANOVA). Three-way ANOVA was used to test the difference between plant species, populations and treatments (temperature, photoperiod, water stress, salinity stress, pH and seed burial depth). Finally, least significant difference test was used as post-hoc to separate the means of dormancy and photoperiod experiments. While a three-parameter sigmoid model was fitted to final germination percentage values obtained from water and salt stress experiments. The model was

1 $${\rm{G}}={{\rm{G}}}_{{\rm{\max }}}/(1+{\rm{e}}\,[-{\rm{x}}-{{\rm{T}}}_{{\rm{50}}})/{{\rm{G}}}_{{\rm{rate}}}]$$

In above model; G is the cumulative percentage germination at time x, G_max_ is the maximum germination (%), T_50_ is osmotic potential or salinity level required for 50% inhibition of maximum germination, and G_rate_ indicates the slope.

Similarly, the final germination percentage values resulting from temperature, pH and seed burial experiments were modelled with a three-parameter Gaussian model. The model was:

2 $${\rm{G}}={\rm{a}}\times {\rm{e}}\,[-0.5-{\{({\rm{x}}{\rm{-}}{\rm{b}}){\rm{/}}{\rm{c}}\}}^{{\rm{2}}}]$$

The Gaussian model gives a “bell curved” graph. In the above model, “a” corresponds to the height of the curve’s peak (maximum germination or emergence); “b” is the position of center of the peak (temperature, pH or depth of seed burial to achieve maximum germination or seedling emergence); and “c” is the width of the “bell”.

## Electronic supplementary material

Supplementary Material
