# Supplementary material for: Germination Biology of Two Invasive Physalis Species and Implications for Their Management in Arid and Semi-arid Regions
Source: Sci Rep. 2017 Dec 5;7:16960. doi: 10.1038/s41598-017-17169-5 (PMC5717255; doi:10.1038/s41598-017-17169-5)
Supplement: Supplementary file 1 — Supplementary Material [file 41598_2017_17169_MOESM1_ESM.doc]

**Germination Biology of Two Invasive *Physalis* Species and Implications for Their Management in** **Arid and Semi-arid Regions**

**Cumali Ozaslan^1^, Shahid Farooq^2#^, Huseyin Onen^2*#^, Selcuk Ozcan^3^, Bekir Bukun^1^, and Hikmet Gunal^4^**

^1^Department of Plant Protection, Dicle University, Diyarbakir, Turkey

^2^Department of Plant Protection, Gaziosmanpasa University, Tokat, Turkey

^3^Pistachio Research Station, General Directorate of Agricultural Research and Policies, Gaziantep, Turkey

^4^Department of Soil Science and Plant Nutrition, Gaziosmanpasa University, Tokat, Turkey

*Corresponding: [onenhuseyin@gmail.com](mailto:onenhuseyin@gmail.com)

^#^These authors contributed equally to this work

**Supplementary Material**

**Table S1.** Three-way ANOVA of invasive plant species (*Physalis angulata* versus *P. philadelphica*), populations (755, 527 and 120 m altitude gradient), seed age (fresh versus 3, 6 and 12 months) and their all possible interactions on final germination percentage

| **Source** | **df** | **SS** | **MS** | ***F value*** | ***P value*** |
| --- | --- | --- | --- | --- | --- |
| **Plant species (S)** | 1 | 15.13 | 15.13 | 1.38 | 0.245^ns^ |
| **Populations (P)** | 2 | 23.86 | 11.93 | 1.09 | 0.344^ns^ |
| **Seed age (T)** | 3 | 50275.49 | 16758.50 | 1533.17 | 0.000* |
| **S × P** | 2 | 15.75 | 7.88 | .720 | 0.492^ns^ |
| **S× T** | 3 | 3.38 | 1.13 | .103 | 0.958^ns^ |
| **P × T** | 6 | 40.47 | 6.75 | .617 | 0.716^ns^ |
| **S × P × T** | 6 | 45.92 | 7.65 | .700 | 0.651^ns^ |
| **Error** | 48 | 524.67 | 10.93 |  |  |

* = individual and interactions are significant at p≤0.01, ^ns^ = individual and interactions are non-significant at p≤0.01, SS = Sum of squares, MS = Mean square

**Table S2.** Three-way ANOVA of invasive plant species (*Physalis angulata* versus *P. philadelphica*), populations (755, 527 and 120 m altitude gradient), seed age (fresh versus 3, 6 and 12 months) and their all possible interactions on final germination percentage of seeds placed under running tap water for 24 hours

| **Source** | **df** | **SS** | **MS** | ***F value*** | ***P value*** |
| --- | --- | --- | --- | --- | --- |
| **Plant species (S)** | 1 | 32.00 | 32.00 | 2.58 | .115^ns^ |
| **Populations (P)** | 2 | 10.11 | 5.06 | 0.41 | .667^ns^ |
| **Seed age (T)** | 3 | 734.67 | 244.89 | 19.77 | .000* |
| **S × P** | 2 | 86.33 | 43.17 | 3.48 | .039** |
| **S× T** | 3 | 68.89 | 22.96 | 1.85 | .150^ns^ |
| **P × T** | 6 | 54.33 | 9.06 | 0.73 | .627^ns^ |
| **S × P × T** | 6 | 42.11 | 7.02 | 0.57 | .755^ns^ |
| **Error** | 48 | 594.67 | 12.39 |  |  |

* = individual and interactions are significant at p≤0.01, ** = individual and interactions are significant at p≤0.05, ^ns^ = individual and interactions are non-significant at p≤0.01, SS = Sum of squares, MS = Mean square

**Table S3.** Three-way ANOVA of invasive plant species (*Physalis angulata* versus *Physalis philadelphica*), populations (755, 527 and 120 m altitude gradient), photoperiod (0, 12 and 24 hours) and their all possible interactions on final germination percentage.

| **Source** | **df** | **SS** | **MS** | ***F* value** | ***P* value** |
| --- | --- | --- | --- | --- | --- |
| **Plant species (S)** | 1 | 3.60 | 3.60 | .474 | .494^ns^ |
| **Populations (P)** | 2 | 15.28 | 7.64 | 1.006 | .371^ns^ |
| **Phototoperiod (L)** | 2 | 2528.35 | 1264.17 | 166.339 | .000* |
| **S × P** | 2 | 28.800 | 14.40 | 1.895 | .158^ns^ |
| **S× L** | 2 | 99.20 | 49.60 | 6.526 | .002** |
| **P × L** | 4 | 16.17 | 4.044 | .532 | .712 ^ns^ |
| **S × P × L** | 4 | 80.00 | 20.00 | 2.632 | .041** |
| **Error** | 72 | 547.20 | 7.60 |  |  |

* = individual and interactions are significant at p≤0.01, ** = individual and interactions are significant at p≤0.05, ^ns^ = individual and interactions are non-significant at p≤0.01, SS = Sum of squares, MS = Mean square

**Table S4.** Three-way ANOVA of invasive plant species (*Physalis angulata* versus *Physalis philadelphica*), populations (755, 527 and 120 m altitude gradient), increasing temperature regimes (5, 10, 15, 20, 25, 30, 35, 40, 45 and 50 ºC) and their all possible interactions on final germination percentage.

| **Source** | **df** | **SS** | **MS** | ***F* value** | ***P* value** |
| --- | --- | --- | --- | --- | --- |
| **Plant species (S)** | 1.00 | 48939.02 | 48939.02 | 1076.11 | 0.00* |
| **Populations (P)** | 2.00 | 425.64 | 212.82 | 4.68 | 0.01* |
| **Temperature regimes (T)** | 5.00 | 64370.31 | 12874.06 | 283.08 | 0.00* |
| **S × P** | 2.00 | 3225.38 | 1612.69 | 35.46 | 0.00* |
| **S× T** | 5.00 | 38286.58 | 7657.32 | 168.37 | 0.00* |
| **P × T** | 10.00 | 6923.69 | 692.37 | 15.22 | 0.00* |
| **S × P × T** | 10.00 | 24893.02 | 2489.30 | 54.74 | 0.00* |
| **Error** | 144.00 | 6548.80 | 45.48 |  |  |
| **Total** | 180.00 | 1036848.00 |  |  |  |

* = individual and interactions are significant at p≤0.01, SS = Sum of squares, MS = Mean square

**Table S5.** Three-way ANOVA of invasive plant species (*Physalis angulata* versus *Physalis philadelphica*), populations (755, 527 and 120 m altitude gradient), different osmotic potentials (0, -0.2, -0.4, -0.6, -0.8, -1.0, -1.2, -1.4 MPa) and their all possible interactions on final germination percentage.

| **Source** | **df** | **SS** | **MS** | ***F* value** | ***P* value** |
| --- | --- | --- | --- | --- | --- |
| **Plant species (S)** | 1 | 2619.16 | 2619.16 | 730.27 | 0.00* |
| **Populations (P)** | 2 | 8642.81 | 4321.40 | 1204.89 | 0.00* |
| **Osmotic potentials (O)** | 7 | 199690.95 | 28527.27 | 7953.960 | 0.00* |
| **S × P** | 2 | 625.38 | 312.69 | 87.18 | 0.00* |
| **S× O** | 7 | 2017.95 | 288.27 | 80.37 | 0.00* |
| **P × O** | 14 | 6826.80 | 487.62 | 135.96 | 0.00* |
| **S × P × O** | 14 | 1128.32 | 80.59 | 22.47 | 0.00* |
| **Error** | 144 | 516.46 | 3.58 |  |  |
| **Total** | 192 | 671527.73 |  |  |  |

* = individual and interactions are significant at p≤0.01, SS = Sum of squares, MS = Mean square

**Table S6.** Three-way ANOVA of invasive plant species (*Physalis angulata* versus *Physalis philadelphica*), populations (755, 527 and 120 m altitude gradient), NaCl concentrations (0, 50, 100, 150, 200, 300, 400 and 600 mM) and their all possible interactions on final germination percentage.

| **Source** | **df** | **SS** | **MS** | ***F* value** | ***P* value** |
| --- | --- | --- | --- | --- | --- |
| **Plant species (S)** | 1 | 58.09 | 58.09 | 14.35 | 0.00* |
| **Populations (P)** | 2 | 2621.74 | 1310.87 | 324.02 | 0.00* |
| **Salinity (Na)** | 6 | 121472.27 | 20245.37 | 5004.33 | 0.00* |
| **S × P** | 2 | 649.19 | 324.59 | 80.23 | 0.00* |
| **S× Na** | 6 | 385.32 | 64.22 | 15.87 | 0.00* |
| **P × Na** | 12 | 2425.91 | 202.16 | 49.97 | 0.00* |
| **S × P × Na** | 12 | 881.11 | 73.42 | 18.15 | 0.00* |
| **Error** | 126 | 509.74 | 4.04 |  |  |
| **Total** | 168 | 823048.70 |  |  |  |

* = individual and interactions are significant at p≤0.01, SS = Sum of squares, MS = Mean square

**Table S7.** Three-way ANOVA of invasive plant species (*Physalis angulata* versus *Physalis philadelphica*), populations (755, 527 and 120 m altitude gradient), pH (4, 5, 6, 7, 8, 9, 10 and 11) and their all possible interactions on final germination percentage.

| **Source** | **df** | **SS** | **MS** | ***F* value** | ***P* value** |
| --- | --- | --- | --- | --- | --- |
| **Plant species (S)** | 1 | 145.91 | 145.91 | 30.22 | 0.00* |
| **Populations (P)** | 2 | 1351.10 | 675.55 | 139.93 | 0.00* |
| **pH (pH)** | 7 | 117904.31 | 16843.47 | 3488.86 | 0.00* |
| **S × P** | 2 | 1340.62 | 670.31 | 138.84 | 0.00* |
| **S× pH** | 7 | 793.59 | 113.37 | 23.48 | 0.00* |
| **P × pH** | 14 | 10884.80 | 777.48 | 161.04 | 0.00* |
| **S × P × pH** | 14 | 1554.97 | 111.07 | 23.00 | 0.00* |
| **Error** | 144 | 695.20 | 4.82 |  |  |
| **Total** | 192 | 620606.78 |  |  |  |

* = individual and interactions are significant at p≤0.01, SS = Sum of squares, MS = Mean square

**Table S8.** Three-way ANOVA of invasive plant species (*Physalis angulata* versus *Physalis philadelphica*), populations (755, 527 and 120 m altitude gradient), seed burial depth (0, 1, 2, 4, 6, 8, 10 and 12 cm) and their all possible interactions on final germination percentage.

| **Source** | **df** | **SS** | **MS** | ***F* value** | ***P* value** |
| --- | --- | --- | --- | --- | --- |
| **Plant species (S)** | 1 | 13.46 | 13.46 | 4.39 | 0.03** |
| **Populations (P)** | 2 | 31.79 | 15.89 | 5.18 | 0.00* |
| **Burial depth (D)** | 7 | 156631.23 | 22375.89 | 7304.72 | 0.00* |
| **S × P** | 2 | 65.93 | 32.96 | 10.76 | 0.00* |
| **S× D** | 7 | 127.24 | 18.17 | 5.93 | 0.00* |
| **P × D** | 14 | 238.80 | 17.05 | 5.56 | 0.00* |
| **S × P × D** | 14 | 201.86 | 14.41 | 4.70 | 0.00* |
| **Error** | 144 | 441.10 | 3.06 |  |  |
| **Total** | 192 | 292607.28 |  |  |  |

* = individual and interactions are significant at p≤0.01, ** = individual and interactions are significant at p≤0.05, SS = Sum of squares, MS = Mean square
